# Removal of an embedded esophageal fishbone by minimal incision using the gel immersion endoscopic ultrasound-guided technique

**DOI:** 10.1055/a-2413-7733

**Published:** 2024-09-25

**Authors:** Shunta Nagamori, Takehide Fukuchi, Shinpei Kondo, Masaki Nishimura, Hayato Yoshimura, Shigeru Iwase, Shin Maeda

**Affiliations:** 1 36993 Department of Gastroenterology, Fujisawa City Hospital, Fujisawa, Japan; 2 26438 Department of Gastroenterology, Yokohama City University School of Medicine Graduate School of Medicine, Yokohama, Japan

Endoscopic removal of ingested esophageal foreign bodies using forceps is a simple procedure 1 , but it can become relatively difficult to identify and remove the foreign body when it is completely embedded in the wall 2 3 4 . Salvage surgery is highly complex and invasive; however, without intervention, there is a risk of perforation and mediastinal abscess formation 5 .

An 82-year-old woman presented to the emergency department with pharyngeal pain on swallowing for 1 day. Computed tomography showed a 25-mm fishbone in the upper esophagus ( Fig. 1 ). We performed outpatient gastroscopy and were initially able to detect the fishbone, but it accidentally strayed into the esophageal wall and was completely lost, leaving no penetration point such as a mucosal hole or erosion. With the patient under general anesthesia, we again attempted to remove the fishbone using miniature-probe endoscopic ultrasound (EUS) with a novel gel immersion technique, which clearly revealed the whole embedded fishbone beneath the mucosa without any evidence of muscular infiltration ( Fig. 2 ). After the fishbone had been accurately located, we performed a local injection and started a mucosal incision, using a DualKnife (KD-650Q; Olympus Medical Systems, Tokyo, Japan), 3 mm from the tip of the bone, which was identified just under the incision line ( Fig. 3 ). The fishbone was removed using a reopenable clip (SureClip; Micro-Tech, Nanjing, China), which enabled precise movement Fig. 4 ). Finally, a synthetic hemostatic material (PuraStat; 3-D Matrix, Tokyo, Japan) was applied to the incision line to prevent bleeding and postoperative stricture formation.

**Fig. 1:**
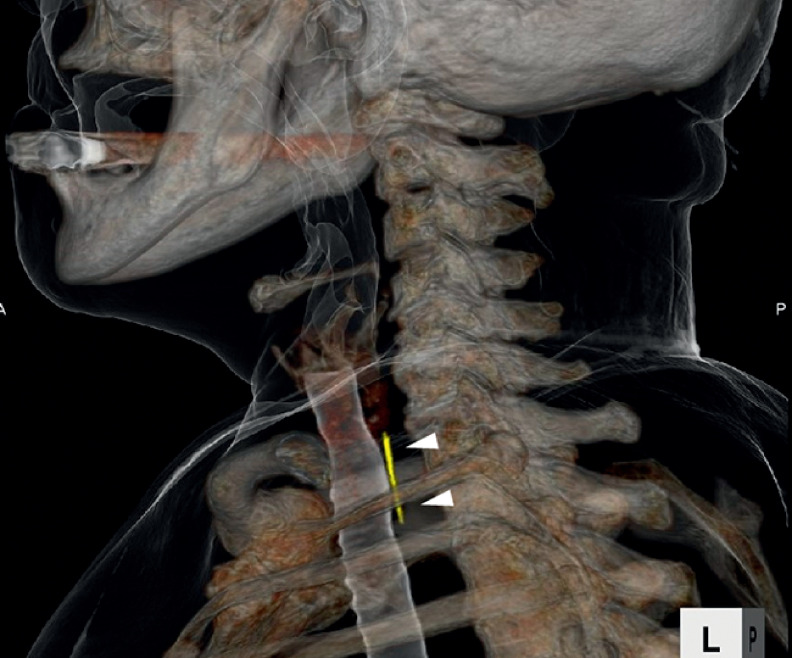
Computed tomography image showing a 25-mm fishbone (arrowheads) in the upper esophagus.

**Fig. 2:**
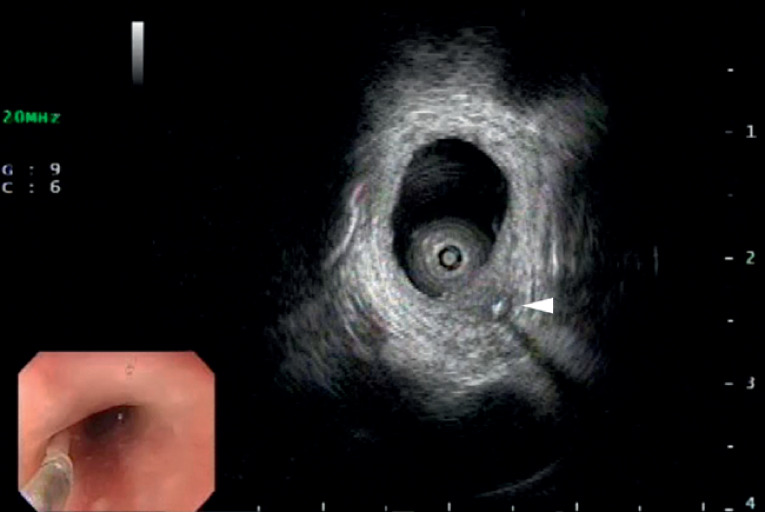
Endoscopic ultrasound image using a miniature probe with a novel gel immersion technique clearly showing the whole embedded fishbone beneath the mucosa.

**Fig. 3:**
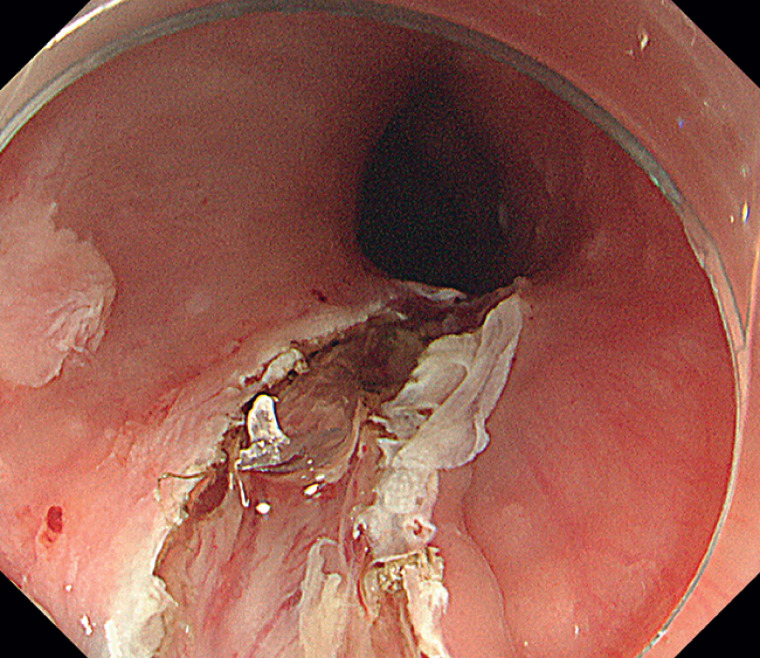
Endoscopic image showing the edge of the fishbone, which was revealed after the initial mucosal incision.

**Fig. 4:**
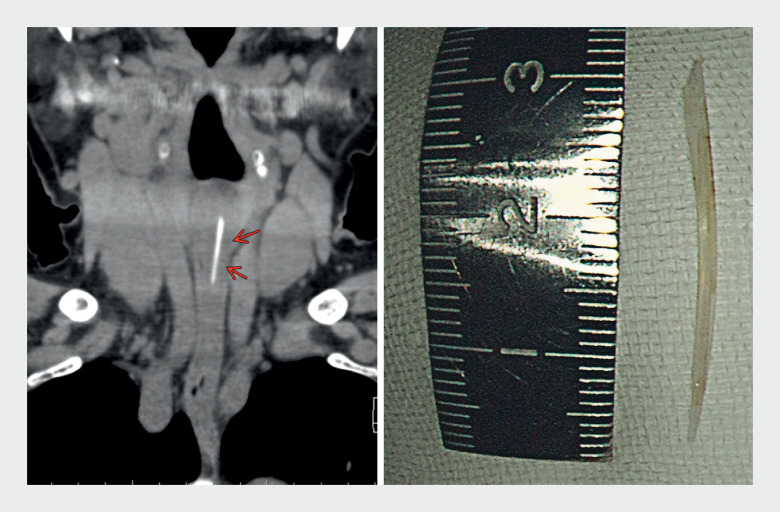
Photograph of the extracted fishbone (right), which is seen to be the same shape as on computed tomography image (left).

In this report, we present the first case in which a fishbone was completely identified using EUS guidance with gel immersion and successfully removed with a minimal incision, without any complications ( Video 1 ).

Removal of an embedded esophageal fishbone by endoscopic minimal incision with the gel immersion endoscopic ultrasound-guided technique.Video 1

Endoscopy_UCTN_Code_TTT_1AO_2AL

## Notes

Endoscopy E-Videos https://eref.thieme.de/e-videos
*E-Videos* is an open access online section of the journal *Endoscopy* , reporting on interesting cases and new techniques in gastroenterological endoscopy. All papers include a high-quality video and are published with a Creative Commons CC-BY license. Endoscopy E-Videos qualify for HINARI discounts and waivers and eligibility is automatically checked during the submission process. We grant 100% waivers to articles whose corresponding authors are based in Group A countries and 50% waivers to those who are based in Group B countries as classified by Research4Life (see: https://www.research4life.org/access/eligibility/ ). This section has its own submission website at https://mc.manuscriptcentral.com/e-videos .
